# Phenome-wide genetic-correlation analysis and genetically informed causal inference of amyotrophic lateral sclerosis

**DOI:** 10.1007/s00439-023-02525-5

**Published:** 2023-02-11

**Authors:** Salvatore D’Antona, Gita A. Pathak, Dora Koller, Danilo Porro, Claudia Cava, Renato Polimanti

**Affiliations:** 1 Institute of Bioimaging and Molecular Physiology, National Research Council, Milan, Italy; 2 Division of Human Genetics, Department of Psychiatry, Yale University School of Medicine, New Haven, CT USA; 3 Veterans Affairs Connecticut Healthcare System, West Haven, CT USA; 4 Department of Genetics, Microbiology and Statistics, University of Barcelona, Barcelona, Catalonia Spain

## Abstract

Leveraging genome-wide association statistics generated from a large study of amyotrophic lateral sclerosis (ALS; 29,612 cases and 122,656 controls) and UK Biobank (UKB; 4,024 phenotypes, up to 361,194 participants), we conducted a phenome-wide analysis of ALS genetic liability and identified 46 genetically correlated traits, such as fluid intelligence score (*r*_*g*_ = − 0.21, *p* = 1.74 × 10^–6^), "spending time in pub or social club” (*r*_*g*_ = 0.24, *p* = 2.77 × 10^–6^), non-work related walking (*r*_*g*_ = − 0.25, *p* = 1.95 × 10^–6^), college education (*r*_*g*_ = − 0.15, *p* = 7.08 × 10^–5^), “ever diagnosed with panic attacks (*r*_*g*_ = 0.39, *p* = 4.24 × 10^–5^), and “self-reported other gastritis including duodenitis” (*r*_*g*_ = 0.28, *p* = 1.4 × 10^–3^). To assess the putative directionality of these genetic correlations, we conducted a latent causal variable analysis, identifying significant genetic causality proportions (gĉp) linking ALS genetic liability to seven traits. While the genetic component of “self-reported other gastritis including duodenitis" showed a causal effect on ALS (gĉp = 0.50, *p* = 1.26 × 10^–29^), the genetic liability to ALS is potentially causal for multiple traits, also including an effect on "ever being diagnosed with panic attacks” (gĉp = 0.79, *p* = 5.011 × 10^–15^) and inverse effects on “other leisure/social group activities” (gĉp = 0.66, *p* = 1 × 10^–4^) and prospective memory result (gĉp = 0.35, *p* = 0.005). Our subsequent Mendelian randomization analysis indicated that some of these associations may be due to bidirectional effects. In conclusion, this phenome-wide investigation of ALS polygenic architecture highlights the widespread pleiotropy linking this disorder with several health domains.

**Supplementary Information:**

The online version contains supplementary material available at 10.1007/s00439-023-02525-5.

## Introduction

Amyotrophic lateral sclerosis (ALS) is a complex neurological disorder due to the degeneration of the upper and lower motor neurons at the bulbar and spinal level (Rowland and Shneider 2001), with an age of onset ranging between 50 and 65 years of age (Oskarsson et al. 2018). Worldwide, ALS incidence is 4.5 cases per 100,000 person-year (Joilin et al. 2019). The disease is characterized by initial muscle weakness that worsens to atrophy, preventing the patient from breathing and swallowing, and eventually causing death (Polkey et al. 1999). Indeed, half of ALS patients die in the first 3 years after diagnosis of the disease and only 20% of them survive in the following 7 years (Talbot 2009).

Familial forms of ALS are due to rare mutations in genes such as *TAR DNA-Binding Protein* (*TARDBP*), *Superoxide Dismutase 1* (*SOD1*), *FUS RNA Binding Protein* (*FUS*), and *C9orf72-SMCR8 Complex Subunit* (*C9orf72*) (Abhinav et al. 2007; Greenway et al. 2006; Ticozzi et al. 2011; Valdmanis and Rouleau 2008). However, more than 90% of ALS cases are not due to monogenic forms, but they are likely due to a complex genetic architecture characterized by the additive effect of many risk loci with small individual effects.

To date, the largest ALS genome-wide association study (GWAS) investigated 29,612 cases and 122,656 controls, identifying 15 risk loci (van Rheenen et al. 2021). These ALS-associated variants were shared with other neurologic traits and diseases, but their effect on the transcriptomic regulation of brain regions and cell types appears to be distinct from other brain disorders (van Rheenen et al. 2021). Beyond gene discovery, genome-wide association statistics can be used to examine ALS genetic liability in the context of other human traits and diseases. Indeed, study designs based on phenome-wide screening are invaluable tools to generate novel hypotheses regarding the genetically shared risk factors and comorbid conditions of ALS (Liu and Crawford 2022). In particular, genetic-correlation (*r*_*g*_*)* analysis can provide insights into the biological pathways and/or the causality relationships linking ALS genetic liability to different health domains (van Rheenen et al. 2019). However, correlation does not prove causation. Accordingly, several methods have been developed to perform causal inference analyses using genetic information as an anchor. Indeed, while observational epidemiological studies can be affected by confounding, reverse causation, and various biases, we can use the association of genetic variants with human traits and diseases to circumvent some of these biases and generate more reliable evidence regarding the cause–effect relationships underlying complex comorbidities. Many causal inference analyses have used the two-sample Mendelian randomization (MR) approach to test the causal effect of the risk factor (i.e., exposure) on the outcome using the associations of genetic variants (i.e., instrumental variable) with exposure and outcome from two sources (Sanderson et al. 2019). Another established method to perform genetically informed causal inference analyses is the latent causal variable (LCV) approach which is based on the assessment of a latent variable that mediates the *r*_*g*_ between two traits of interest (O'Connor and Price 2018).

In the present study, leveraging the genome-wide association statistics from the largest ALS study (van Rheenen et al. 2021), we conducted a phenome-wide *r*_*g*_ analysis with respect to 957 traits with strong SNP-based heritability estimates (z score > 4) available from the UK Biobank (UKB) (Bycroft et al. 2018). Considering ALS genetically correlated traits, we performed causal inference analyses using LCV and MR approaches. These methods are based on different assumptions and effects consistent between them are less likely to be biased by violations of the assumptions underlying each model. We considered results consistent across these two methods to be reliable evidence of possible cause–effect relationships related to ALS pathogenesis.

## Materials and methods

To investigate ALS genetic liability in the context of other human traits and diseases, we designed the present work by applying complementary analytic approaches to multiple large-scale genome-wide datasets (Supplemental Fig. 1). This study was conducted using genome-wide association statistics generated by the previous studies. Owing to the use of previously collected, deidentified, aggregated data, this study is exempting from institutional review board approval.

### Data sources

We investigated data from the largest GWAS of ALS (van Rheenen et al. 2021) that included 29,612 cases and 122,656 controls (91% of European descent and 9% of East Asian descent) (van Rheenen et al. 2021). With respect to other traits and diseases, we used genome-wide association statistics generated from the UKB (Bycroft et al. 2018). This cohort includes approximately 500,000 individuals (54% females) from the United Kingdom with an age at recruitment between 37 and 73 (Bycroft et al. 2018). UKB participants were assessed for a wide range of outcomes including diet, cognitive function, work history, health status, and other relevant phenotypes. Genome-wide data were also generated in this cohort. Details regarding UKB phenotypic and genetic assessment have been previously described (Bycroft et al. 2018). In our analysis, we used genome-wide association statistics generated from the analysis of 361,194 UKB participants of European descent. Specifically, we estimated SNP-heritability for 4024 UKB phenotypes (Supplemental Table 1) and analyzed with respect to ALS only those with SNP-heritability z > 4 (Supplemental Table 2; see SNP-based Heritability and Genetic Correlation). Details regarding the quality control and the association analysis are available at https://github.com/Nealelab/UK_Biobank_GWAS. Briefly, the genome-wide association analysis was conducted using regression models available in Hail (available at https://github.com/hail-is/hail) and including the top-20 within-ancestry principal components (PC), sex, age, age^2^, sex × age, and sex × age^2^ as covariates. We also analyzed sex-stratified genome-wide association statistics from UKB to investigate possible differences between females and males in the pleiotropy between ALS and other phenotypes. The sex-specific covariates included the top-20 PCs, age, and age^2^. ALS and UKB genome-wide association statistics were processed by removing variants with a minor allele frequency (MAF) < 1%. With respect to UKB genome-wide association statistics generated from case–control phenotypes, we also removed variants with a minor allele count < 20 in the smaller group between cases and controls. To verify the specificity of the findings identified with respect to ALS, we also analyzed genome-wide association statistics generated from 111,326 clinically diagnosed/'proxy' Alzheimer’s disease cases and 677,663 controls (Bellenguez et al. 2022).

### SNP-based heritability and genetic correlation

We calculated the SNP-based heritability for ALS using genome-wide data available from a previous study (van Rheenen et al. 2021) and for the UKB phenotypes (Supplemental Table 1). For this analysis, we used the linkage disequilibrium score regression (LDSC) (Bulik-Sullivan et al. 2015) testing 1,217,311 SNPs present in the HapMap 3 reference panel and applying the pre-computed LD scores based on 1000 Genomes Project reference data on individuals of European ancestry (available at https://github.com/bulik/ldsc). We decided to use European-ancestry LD scores, because the ALS GWAS was generated from a sample including > 90% individuals of European descent. In line with the recommendations of the LDSC developers (Bulik-Sullivan et al. 2015), we tested ALS *r*_*g*_ with UKB traits with SNP-based heritability *z* score > 4. Additionally, we also performed a sex-stratified genetic-correlation analysis, estimating statistical differences between female and male rg estimates using a *z* test. To account for multiple testing, we considered statistically significant *r*_*g*_s as those surviving a 5% false discovery rate (FDR) correction (FDR *Q* value < 0.05).

### Polygenic risk scoring among ALS datasets

To verify the transferability of genetic effects between the ALS GWAS meta-analysis (van Rheenen et al. 2021) and ALS cases in the UKB cohort (G6_ALS: motor neuron disease), we conducted polygenic risk score analysis on the basis of the genome-wide association statistics using the gtx R package incorporated in PRSice software (Euesden et al. 2015). The PRS was calculated considering a p value-informed clumping with an LD cutoff of *R*2 = 0.01 within a 250-kilobase window, excluding the major histocompatibility complex region of the genome because of its complex LD structure and including only variants with a minor allele frequency greater than 1%. The European samples from the 1000 Genomes Project were used as the LD reference panel. To maximize the variance explained, we considered multiple p value threshold (PT = 5 × 10^–8^, 1 × 10^–7^, 1 × 10^–6^, 1 × 10^–5^, 1 × 10^–4^, 0.001, 0.1, 0.05, 0.3, 0.5, 1).

### Genetically informed causal inference analysis

To identify causative effects underlying the *r*_*g*_s observed, we applied the LCV approach (O'Connor and Price 2018). Under the assumption of a single effect-size distribution, LCV examines the presence of a single latent trait between genetically correlated traits to estimate a genetic causality proportion (gĉp), ranging from 0 to 1. Values near 0 indicate partial causality, while values approaching 1 indicate full causality (O'Connor and Price 2018). Positive and negative gĉp values reflect the direction of the putative causal effect (i.e., phenotype #1 → phenotype #2 and phenotype #2 → phenotype #1, respectively). To avoid confusion, we describe the LCV effects oriented in accordance with positive gĉp values. Information regarding the sign of the LCV effect is provided by the LCV rho statistics: rho > 0 corresponds to a positive effect, while rho < 0 corresponds to a negative effect. The LCV analysis was performed in R using the European-ancestry LD scores. The primary LCV analysis was conducted using the sex-combined UKB genome-wide association statistics, and subsequently, the traits identified were tested further using sex-stratified UKB genome-wide association statistics.

To validate the LCV results with an independent method, we conducted a bidirectional two-sample MR analysis. Indeed, LCV and MR approaches are based on different modeling approaches. Accordingly, estimates concordant between these two methods have to be robust to different assumptions.

While the LCV method estimates causal effects using genome-wide information, the MR approach infers putative causal relationships between exposure and outcome using a limited number of SNPs as instrumental variables (Zheng et al. 2017). Additionally, MR assumptions are: (i) the instrumental variables (genetic variants) are associated with the exposure, (ii) the genetic variants are not associated with confounders related to exposure and outcome, and (iii) the genetic variants affect the outcome only through the exposure factor (Davies et al. 2018).

In the present study, the MR analysis was conducted using the TwoSampleMR package (Hemani et al. 2017) within the R environment. This R package permits to perform a wide range of MR analyses using genome-wide association statistics. With respect to MR approaches based on individual-level data, statistics-level MR analyses can be used to integrate sample sizes and assessments that may be present across different data sources. For each MR test, we defined the instrumental variable based on LD-independent variants (*r*^2^ < 0.001 within a 10,000-kilobase window) based on the 1000 Genomes Project Phase 3 reference panel for European populations. The clumping procedure was conducted considering a *p* value of 10^–5^ for the exposure GWAS. Similar to previous MR studies (Bountress et al. 2021; Polimanti et al. 2019; Tylee et al. 2022; Wendt et al. 2019), we used this threshold to maximize the statistical power of the analyses performed. The analysis was conducted using five MR methods: inverse variance weighted (IVW), MR-Egger, simple mode, weighted median, and weighted mode (Hemani et al. 2018). These different methods allowed us to verify whether the estimates observed were robust to the sensitivities unique to each of the stated methods. We referred to the IVW method as the primary method due to its higher statistical power (Bowden et al. 2017). As recommended for genetically informed causal inference analyses (Sterne and Davey Smith 2001), we avoided inference based simply on p value thresholds. The direction and strength of the effects estimated via LCV and MR analyses, together with the corresponding p values, were considered to better reflect the spectrum of evidence related to the LCV and MR results. To assess possible biases due to potential confounders (e.g., horizontal pleiotropy and heterogeneity), we performed multiple sensitivity analyses, including MR-PRESSO (Pleiotropy RESidual Sum and Outlier) global test (Verbanck et al. 2018), heterogeneity test (Burgess et al. 2017), and the MR-Egger intercept analysis (Burgess and Thompson 2017).

## Results

**Fig. 1:**
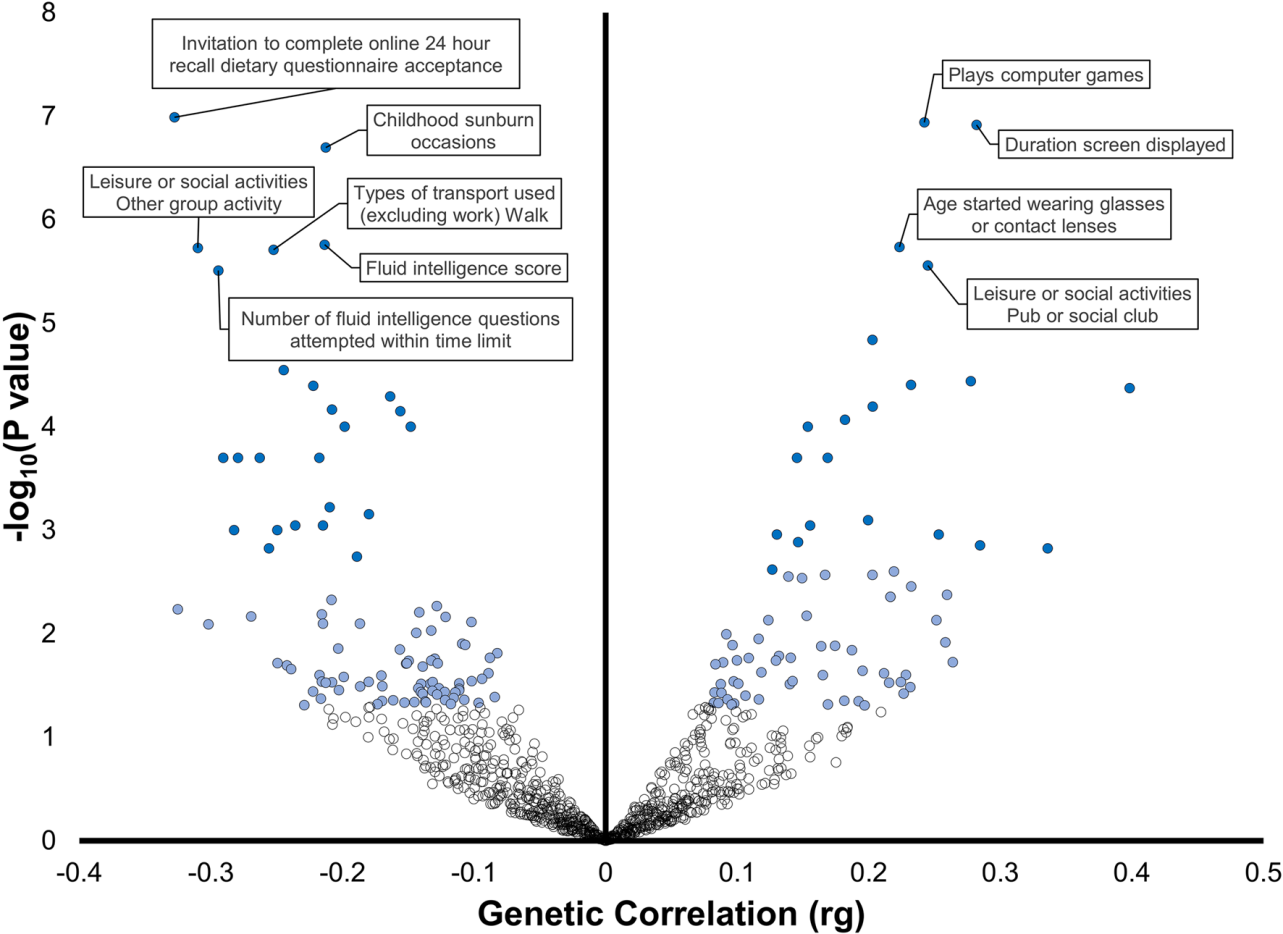
Phenome-wide genetic-correlation analysis. The color of the dots corresponds to the significance strength of the genetic correlations (rg): white (*p* > 0.05), light blue (*p* < 0.05), and blue (FDR *q* < 0.05). Phenotype labels are included for the top ten results

Applying the LDSC method, we calculated ALS SNP-based heritability based on a previously generated GWAS meta-analysis (van Rheenen et al. 2021) and phenotypic traits available from UKB (see Data Sources). ALS SNP-heritability on the observed scale was 3% (observed-scale SNP-*h*^2^ = 0.0357, SE = 0.0039, *z* = 9.15). For the 4,024 traits available from UKB, we estimated the SNP-based heritability and considered 957 phenotypes with an SNP-based heritability *z* score > 4 (Supplemental Table 2) for further analyses as recommended by the LDSC developers (Bulik-Sullivan et al. 2015). These included a wide range of outcomes related to different phenotypic domains such as “qualification: college or university degree” (SNP-*h*^2^ = 0.16, SE = 0.0045, *z* = 35.56), body mass index (SNP-*h*^2^ = 0.23, SE = 0.0068, *z* = 33.82), forced expiratory volume in one second (FEV1; SNP-*h*^2^ = 0.177, SE = 0.006, *z* = 29.5), and hand grip strength (left hand; SNP-*h*^2^ = 0.11, SE = 0.0039, *z* = 28.21). Since UKB has a prevalence similar to the one observed in the UK population (0.05% vs. 0.07%, respectively), there are only a limited number of ALS cases in the UKB (*N* = 167). Accordingly, ALS assessed in UKB (G6_ALS: Motor neuron disease) did not show a statistically significant SNP-h^2^ heritability and was not tested in the genetic-correlation analysis. Nevertheless, we demonstrated that a PRS derived from the ALS GWAS meta-analysis (van Rheenen et al. 2021) is statistically associated with ALS assessed in the UKB cohort (*R*^2^ = 1.97%, *p* = 1.32 × 10^–4^; Supplemental Table 3). Considering the 957 phenotypes with an SNP-based heritability *z* score > 4, ALS genetic-correlation analysis identified evidence of genetic overlap surviving multiple testing correction for 46 of them (FDR *q* < 0.05; Fig. 1; Supplemental Table 4). No statistically significant differences (*p* > 0.05) were observed in the *r*_*g*_ estimates of these traits calculated using sex-stratified UKB genome-wide association statistics (Supplemental Table 5). Ten of the ALS significant genetic correlations were related to traits derived from cognitive function tests. Specifically, ALS genetic liability was negatively correlated with fluid intelligence score (*r*_*g*_ = − 0.21, *p* = 1.74 × 10^–6^) and items related to its assessment (e.g., number of fluid intelligence questions attempted within time limit, *r*_*g*_ = − 0.29, *p* = 3.09 × 10^–6^). Additionally, ALS genetic liability was positively correlated with two items related to the prospective memory test (i.e., duration screen displayed, *r*_*g*_ = 0.28, *p* = 1.21 × 10^–7^; number of attempts, *r*_*g*_ = 0.28, p = 3.63 × 10^–5^) and one related to the pair-matching test (time to complete round, *r*_*g*_ = 0.15, *p* = 1 × 10^–4^). Four ALS genetic correlations were related to leisure and social activities. Specifically, ALS genetic liability was positively correlated with spending time in pub or social club (*r*_*g*_ = 0.24, *p* = 2.77 × 10^–6^), but it was inversely correlated with being part of religious group (*r*_*g*_ = − 0.22, *p* = 4.03 × 10^–5^), attending adult education classes (*r*_*g*_ = − 0.24, *p* = 2.83 × 10^–5^), and other group activities (*r*_*g*_ = − 0.31, *p* = 1.87 × 10^–6^). We also identified two FDR-significant genetic correlations related to non-work-related transportation choices where ALS genetic liability was negatively correlated to walking (*r*_*g*_ = − 0.25, *p* = 1.95 × 10^–6^) and using public transportation (*r*_*g*_ = − 0.20, *p* = 6 × 10^–4^). With respect to education, ALS genetic liability was positively correlated with not having any qualification (*r*_*g*_ = 0.13, *p* = 1.1 × 10^–3^) and negatively genetically correlated with having college education (*r*_*g*_ = − 0.15, *p* = 7.08 × 10^–5^), secondary education (*r*_*g*_ = − 0.16, *p* = 5.08 × 10^–5^), and other professional qualifications (*r*_*g*_ = − 0.20, *p* = 1 × 10^–4^). As also mentioned in the original ALS GWAS (van Rheenen et al. 2021), ASL genetic correlation with educational attainment may be influenced by ascertainment bias in ALS GWAS study that included more educated participants than those usually screened in clinical ascertainment of ALS cases.

We also observed that ALS genetic liability was correlated with multiple mental health outcomes such as "ever thought that life was not worth living” (*r*_*g*_ = − 0.21, *p* = 2 × 10^–4^), “ever contemplated self-harm” (*r*_*g*_ = − 0.29, *p* = 2 × 10^–4^), and “ever diagnosed with panic attacks” (*r*_*g*_ = 0.39, *p* = 4.24 × 10^–5^). With respect to physical health, we identified genetic correlations related to self-reported other gastritis including duodenitis, *r*_*g*_ = 0.28, *p* = 1.4 × 10^–3^). Finally, we also observed that ALS genetic liability was negatively correlated to items related to participation in the UKB dietary questionnaire: acceptance of the invitation to complete online 24-h recall dietary questionnaire (*r*_*g*_ = − 0.32, *p* = 1.02 × 10^–7^) and number of diet questionnaires completed (*r*_*g*_ = − 0.27, *p* = 2 × 10^–4^).

**Fig. 2:**
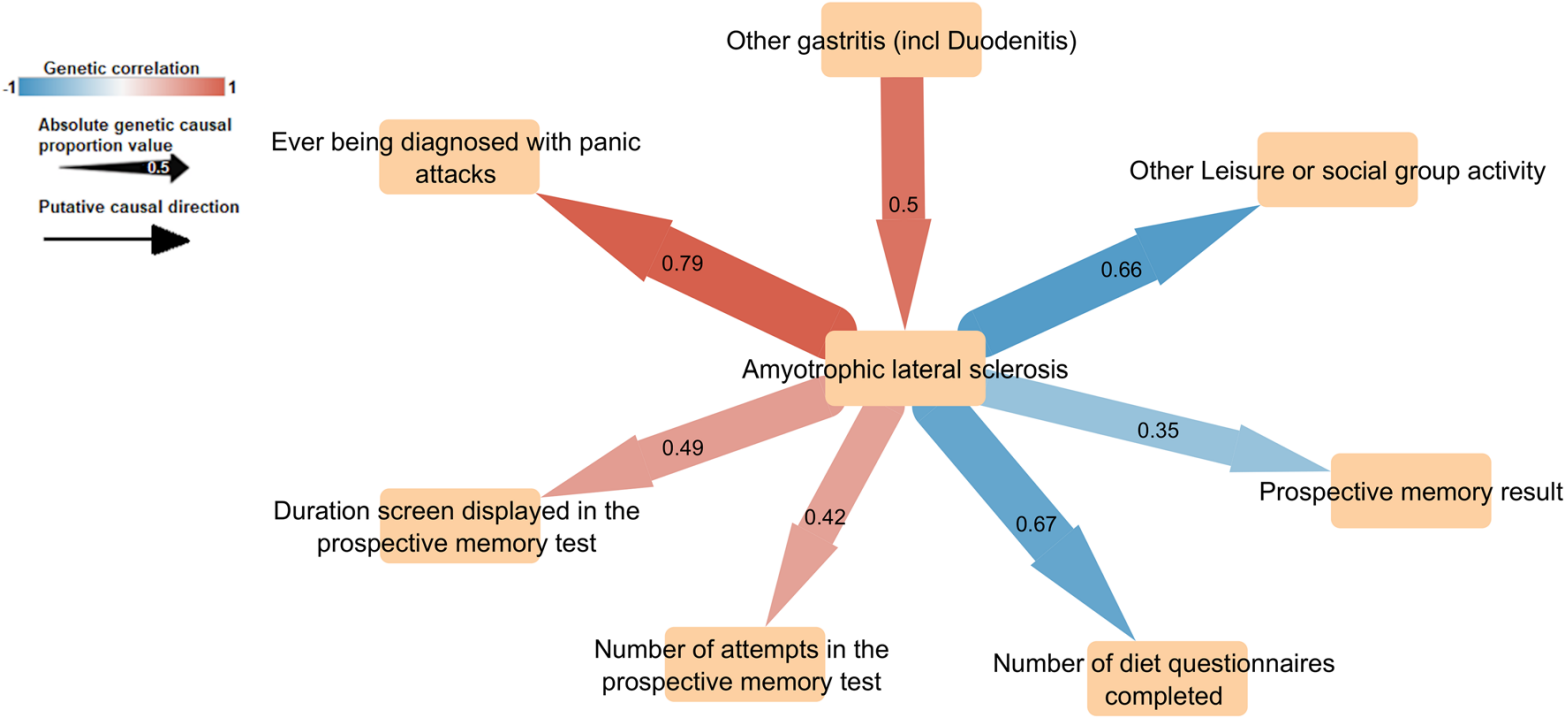
Putative causal effects linking amyotrophic lateral sclerosis with genetically correlated traits phenotypes using the latent causal variable (LCV) approach. The color of arrows refers to the genetic correlation, while the width reflects the genetic causal proportion (gĉp), as an absolute value. The gĉp absolute values are indicated on each arrow

To test causal effects and shared genetic mechanisms underlying ALS genetically correlated traits, we performed a genetically informed causal inference analysis using the LCV approach (O'Connor and Price 2018). As mentioned in the methods, in the LCV analysis, positive and negative gĉp values reflect the direction of the putative causal effect (i.e., phenotype #1 → phenotype #2 and phenotype #2 → phenotype #1, respectively), while the sign of the effect is given by the rho statistics. After FDR multiple testing correction accounting for the number of phenotypes tested *(N* = 46, FDR *q* < 0.05), we identified putative causal effects linking ALS genetic liability to seven of the genetically correlated traits (Fig. 2; Supplemental Table 6). Considering only those traits with genome-wide association statistics were powerful enough to perform LCV analyses in both sexes, no differences (*p* > 0.05) were observed in the gcp estimates when testing sex-stratified UKB data (Supplemental Table 7). Among the traits identified in the sex-combined LCV analysis, only in one instance, the genetic liability of a trait (i.e., on reporting a diagnosis of “other gastritis including duodenitis”) showed a positive causal effect on ALS (gĉp = 0.50, *p* = 1.26 × 10^–29^; rho = 0.28, SE = 0.09). The other LCV results were related to putative causal effects of the genetic liability to ALS on other traits. Three of these showed positive effects of ALS genetic liability (LCV rho > 0): ever being diagnosed with panic attacks (gĉp = 0.79, *p* = 5.011 × 10^–15^; rho = 0.32, SE = 0.12), duration screen displayed in the prospective memory test (gĉp = 0.49, *p* = 6.309 × 10^–7^; rho = 0.19, SE = 0.10), and number of attempts in the prospective memory test (gĉp = 0.42, *p* = 0.001; rho = 0.18, SE = 0.11). Conversely, other three traits appear to be negatively affected by ALS genetic liability (LCV rho < 0): number of diet questionnaires completed (gĉp = 0.67, *p* = 7 × 10^–4^; rho = − 0.26, SE = 0.08), other leisure/social group activities (gĉp = 0.66, *p* = 1 × 10^–4^; rho = − 0.29, SE = 0.07), and prospective memory result (gĉp = 0.35, *p* = 0.005; rho = − 0.17, SE = 0.10).

To understand better the pleiotropy linking the traits identified by the LCV analyses, we performed a genetic-correlation analysis among them. After multiple testing correction (FDR *q* < 0.05), we identified significant genetic correlations among 18 phenotype pairs (Supplemental Table 8). In line with the fact that they are part of the same construct, the strongest genetic correlation (|rg|> 0.89) was observed among items related to prospective memory tests (i.e., number of attempts, duration screening displayed, and prospective memory results). The prospective-memory items showed a strong genetic overlap with the number of diet questionnaires completed (e.g., duration screen displayed rg = − 0.63, *p* = 1.66 × 10^–34^) and leisure/social group activities (e.g., duration screen displayed rg = − 0.44, *p* = 2.95 × 10^–24^). Another strong genetic correlation was observed between panic attacks and “other gastritis including duodenitis” (rg = 0.56, *p* = 2.23 × 10^–6^). The remaining phenotype pairs showed a lower extent of genetic correlation (∣rg∣ < 0.4). Additionally, we also verified the specificity of the LCV-identified traits for ALS with respect to AD. Although there is a partial shared genetic correlation between ALS and AD (rg = 0.25, *p* = 1 × 10^–4^), the only LCV-identified trait for ALS that is also genetically correlated with AD at a nominal significance level was “Duration screen displayed” during the prospective memory test (rg = 0.11, *p* = 0.01; Supplemental Table 9).

Finally, to validate the causal effects identified using the LCV approach, we performed a two-sample bidirectional MR analysis (Supplemental Table 10). LCV and MR are based on different assumptions (Davies et al. 2018; O'Connor and Price 2018). Accordingly, comparing the results obtained from these two methods permitted us to gain additional insights into the pleiotropic genetic mechanisms linking ALS to its genetically correlated traits. Among the causal effects identified by the LCV, we identified a consistent MR result with respect to a very small effect of the genetic liability to other leisure/social group activities on ALS (IVW beta = − 0.006, SE = 0.002). Conversely, our MR analysis showed a bidirectional relationship between the duration of screen displayed in the prospective memory test and ALS genetic liability (duration screen displayed → ALS: IVW beta = 0.230, SE = 0.09; ALS genetic liability → duration screen displayed: IVW beta = 0.033, SE = 0.01), between the number of attempts in the prospective memory test and ALS genetic liability (number of attempts → ALS: IVW beta = 0.59, SE = 0.26; ALS genetic liability → number of attempts: IVW beta = 0.016, SE = 0.003) and between the prospective memory result and genetic liability of ALS (prospective memory result → ALS: IVW beta = 0.51, SE = 0.22; ALS genetic liability → prospective memory result: IVW beta = 0.017, SE = 0.005). We also observed a significant effect of the genetic liability to the number of diet questionnaires completed on ALS (IVW beta = − 0.32, SE = 0.14). This effect was characterized by heterogeneity among the effects of the variants included in the instrumental variable (IVW *Q* = 39.4, *df* = 25, *p* = 0.033). However, we did not identify an effect in the opposite direction (IVW beta = − 0.013, SE = 0.009) or bias due to horizontal pleiotropy (MR-Egger intercept = − 0.0122, *p* = 0.248).

## Discussion

ALS is a complex neurological disorder related to the degeneration of the upper and lower motor neurons (Oskarsson et al. 2018; Rowland and Shneider 2001). Its sporadic form, the most common, is due to a complex genetic architecture characterized by the additive effect of many risk loci with small individual effects. Consistent with the missing heritability observed for complex traits (Genin 2020), we observed that the SNP-based heritability assessed from GWAS data (3%) was much lower than the heritability estimated by previous family-based analyses (~ 50%; Ryan et al. 2019; Trabjerg et al. 2020). Nevertheless, the previous studies showed ALS polygenic risk overlap with schizophrenia, educational attainment, tobacco smoking, and several other traits assessed in large GWAS meta-analyses (Bandres-Ciga et al. 2019; Restuadi et al. 2022; van Rheenen et al. 2021). In the present study, we used genome-wide association statistics available from UKB to investigate ALS genetic liability in the context of a wider range of human traits and diseases. Through this phenome-wide screening approach, we identified putative causal effects and genetic mechanisms shared between ALS and other health outcomes. Our genetic-correlation analysis showed that the polygenic architecture of ALS is correlated with traits related to different domains. In line with the enrichments for brain regions and cells observed in the ALS GWAS (van Rheenen et al. 2021), we observed ALS genetic correlations with multiple traits related to cognitive function and educational attainment. Previous GWAS of educational attainment and related phenotypes showed how their genetic effects have a pattern of enrichments related to brain regions and cells with stronger evidence related to neurons rather than to astrocytes and oligodendrocytes (Okbay et al. 2022). Regarding cognitive functions, we observed ALS genetic correlations with phenotypes related to fluid intelligence (i.e., the ability to generate, transform, and manage different types of new information, in real time) and prospective memory (i.e., the ability to remember to perform learned actions, in the future). Additionally, our genetically informed causal inference analysis showed that ALS genetic liability may affect prospective memory.

ALS patients can manifest a series of cognitive impairments, including abstract reasoning (Elamin et al. 2013). There is still a debate whether cognitive impairment is an early ALS symptom, or a condition related to the long-term consequence of the disease progression (Bersano et al. 2020; Elamin et al. 2013). Our findings indicate that ALS polygenic risk could have a direct effect on cognitive function, supporting that cognitive impairment is an early symptom of the disease.

Another domain with multiple ALS genetically correlated traits is related to the assessment of dietary habits. Although diet is a factor that has been reported to alter ALS risk (Pape and Grose 2020), our findings appear to indicate a more complex scenario. Indeed, while ALS genetic liability is correlated with cheese intake, semi-skimmed milk preference, dried fruit intake, and red wine intake, we also observed a putative causal effect of the genetic liability to ALS to the “number of diet questionnaire complete”. Studies in UKB cohort showed that participation in optional components such as the diet questionnaire is associated with loci associated with educational attainment and Alzheimer’s disease (Tyrrell et al. 2021). Additionally, dietary patterns assessed in UKB participants appear to be causally influenced by factors correlated with education (Cole et al. 2020). Considering these previous findings and our current results, we believe that the study of diet as a possible factor to modify ALS risk should carefully account for the relationship of ALS with cognitive function and educational attainment.

Several ALS genetically correlated traits were related to leisure or social activities. In most of the cases, there was a negative association where ALS genetic effects were correlated with reduced leisure or social activities in different contexts (i.e., group activities, adult education classes, and religious groups). Additionally, the genetic liability to ALS appears to have a possible causal effect on participating in leisure group activities. This observation is in line with previous evidence reporting that cognitively intact ALS patients manifest impairments in social skills, manifesting as an inability to recognize others' emotions and a change in behavior toward apathy (Girardi et al. 2011). Although our findings highlight a general pattern of reduced social activities with respect to increased genetic risk of ALS, we also observed an ALS positive genetic correlation with leisure or social activities in pubs or social clubs, which is consistent with the genetic correlation mentioned previously with red wine intake. The relationship between ALS and alcohol consumption is unclear with a recent large case–control study reporting a non-significant association (D'Ovidio et al. 2019). However, similar to other conditions, the study of alcohol consumption can be biased by misreports and longitudinal changes (Xue et al. 2021). Accordingly, further studies accounting for these possible confounders are needed to understand the potential effect of alcohol consumption on ALS.

Another aspect highlighted by our phenome-wide analysis is the genetic overlap between ALS and mental health. Specifically, we observed genetic correlation with severe psychiatric traits, including "ever thought that life was not worth living”, “ever contemplated self-harm”, and “ever diagnosed with panic attacks”. The latter genetic relationship appears to be due to a potential causal effect of the genetic liability to ALS on the risk of panic attacks. Several studies highlighted the increased risk of ALS patients to be at increased risk of being diagnosed with anxiety, depression, and other internalizing disorders (Caga et al. 2019). Our findings support that the psychiatric comorbidity of ALS could be due to shared genetic mechanisms in addition to the negative impact of ALS on the quality of life for the affected individuals.

Among the putative causal effects identified in our genetically informed causal inference, we observed that only the genetic liability to gastritis could be associated with increased ALS risk. This result was specifically related to a phecode related to gastritis and duodenitis [i.e., other gastritis (incl Duodenitis)]. Other gastritis phenotypes were also available in UKB, but for most of them, the number of cases was much lower than the one observed for gastritis–duodenitis phecode (< 1,800 vs. 10,518, respectively). Another gastritis–duodenitis UKB phenotype (i.e., ICD-10 K29 Gastritis and duodenitis) with a similar number of cases is available and it showed a genetic correlation with ALS that was just outside the significance threshold defined for multiple testing correction (rg = 0.23, *p* = 0.004, FDR *q* = 0.064). This less informative phenotype was based on a single ICD code rather than a phecode combining information from multiple ICD codes. Although this putative causal effect is supported only by the LCV method and not the MR analysis, we speculate that this may be related to the interplay among gut microbiota and the enteric neuro-immune system as a pathogenic path to ALS and other neurodegenerative diseases (Pellegrini et al. 2018). In this context, our findings could point toward the effect of gastritis on microbiome composition (Nardone and Compare 2015) as a novel area for the exploration of ALS pathogenicity.

Our study has four main limitations to consider. Similar to the majority of large-scale genetic studies, we investigated genome-wide data generated mostly from individuals of European descent because of the lack of large cohorts representative of other ancestry groups. Accordingly, further studies in more diverse datasets will be needed to assess the generalizability of the associations identified in the present study. Another limitation of our study is related to the MR analysis. Indeed, because of the limited number of variants reaching genome-wide significance, we had to include suggestive loci in the instrumental variables tested. This has likely limited the statistical power of our MR analysis and may have contributed to the limited concordance between LCV and MR results. We performed sex-stratified genetic correlation and LCV analyses that did not show statistically significant differences in ALS pleiotropy between males and females. However, this analysis should be considered as very preliminary, because it was conducted testing sex-stratified UKB genome-wide association statistics with respect to sex-combined ALS genome-wide association statistics due to the lack of sex-specific ALS GWAS. Finally, our UKB-based analyses were underpowered to investigate phenotypes with low prevalence in the general population. Accordingly, we were not able to follow up findings from studies that focused on uncommon conditions (e.g., schizophrenia, Restuadi et al. 2022) or on traits assessed in large GWAS meta-analyses (e.g., tobacco smoking, Bandres-Ciga et al. 2019).

In conclusion, our phenome-wide study highlighted mechanisms by which ALS genetic liability may be linked to different health domains. In particular, the present findings (i) contributed to the understanding of the possible directionality linking ALS genetic liability to cognitive function, (ii) highlighted the need for further studies to assess the impact of alcohol consumption and dietary habits on ALS risk, and (iii) opened a new possible direction in the study of ALS genetics.

## Supplementary Information

Below is the link to the electronic supplementary material.

Supplementary file1 (TIFF 1024 KB)

Supplementary file2 (XLSX 223 KB)

Supplementary file3 (XLSX 81 KB)

Supplementary file4 (XLSX 14 KB)

Supplementary file5 (XLSX 63 KB)

Supplementary file6 (XLSX 18 KB)

Supplementary file7 (XLSX 12 KB)

Supplementary file8 (XLSX 14 KB)

Supplementary file9 (XLSX 13 KB)

Supplementary file10 (XLSX 10 KB)

Supplementary file11 (XLSX 21 KB)

## Data Availability

Data supporting the findings of this study are available within this article and its additional files.
