# Supplementary figures and images for: Phenome-wide genetic-correlation analysis and genetically informed causal inference of amyotrophic lateral sclerosis
Source: Hum Genet. 2023 Feb 11;142(8):1173–83. doi: 10.1007/s00439-023-02525-5 (PMC10449723; doi:10.1007/s00439-023-02525-5)

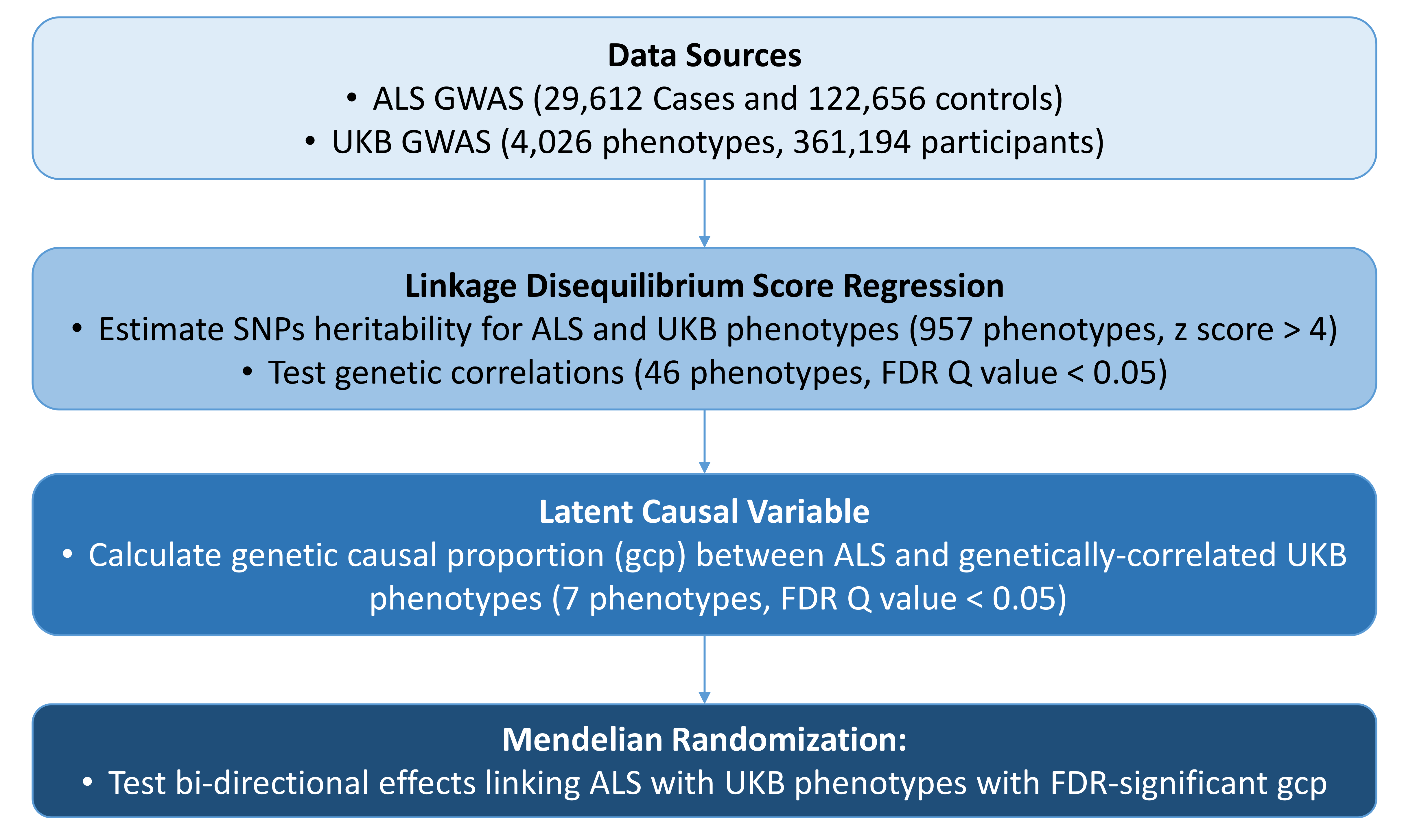

Supplement: Supplementary file 1 — Supplementary file1 (TIFF 1024 KB) [file 439_2023_2525_MOESM1_ESM.tiff]
